# Supplementary material for: Functional decomposition of metabolism allows a system-level quantification of fluxes and protein allocation towards specific metabolic functions
Source: Nat Commun. 2023 Jul 13;14:4161. doi: 10.1038/s41467-023-39724-7 (PMC10345195; doi:10.1038/s41467-023-39724-7)
Supplement: Supplementary file 3 — Description of Additional Supplementary Files - NEW [file 41467_2023_39724_MOESM3_ESM.pdf]

Description of Additional Supplementary Files:

Supplementary Data 1. Set of 3 modified iML1515 models with condition-dependent biomass composition, provided as .json or .mat files, zipped. These correspond to *E. coli* K-12 strain NCM3722 under glucose minimal MOPS media (reference condition, growth rate = 0.96/h), under glucose minimal MOPS media with chloramphenicol treatments (R-limitation, growth rate = 0.35/h), and glucose-limited MOPS growth (C-limitation, growth rate = 0.33/h). In these models, the biomass reactions is not modified compared to the original; instead, sink reactions are added for each individual biomass component, and their flux is constrained depending on the growth condition. Similarly, the acetate excretion flux and the ATP maintenance flux (ATPM) are constrained as described in Fig. 2, panels b and d.

Supplementary Data 2. Experimentally determined growth rates and metabolic exchange fluxes from this work and literature data. See the Methods for a description of the experimental procedures. Growth rates and metabolic fluxes were used to constrain the metabolic models across conditions using the conversion factor $1\ \mathrm{OD_{600}L} = 0.5\mathrm{g_{dw}}$ (Basan et al. 2015).

Supplementary Data 3. The .xlsx file includes fluxes and the functional decomposition of each reaction and protein into the functional component for the 3 FBA models in Supplementary Data 1. For each model, we include the raw (non-coupled) flux modes, the post-coupling flux modes, the flux components, the functional decomposition of each reaction, functional decomposition for all protein-coding genes (mapped as described in Supplementary Note 5) and the functional decomposition of the proteome using matching data from Mori et al., 2021 and Wu, Mori et al., 2023.

Supplementary Data 4. The .xlsx file includes fluxes and (post-coupling) flux modes for anaerobic growth on glucose at two different growth rates, as well as aerobic growth on a variety of carbon sources; for the latter, growth rates and acetate flux data from Basan et al., 2015 (reported in Supplementary Data 2) were used to constrain the acetate excretion fluxes in the FBA calculations, for which we used the same biomass composition and ATP maintenance flux as used in C-limited growth at the same growth rate. For growth on acetate, only the growth rate was constrained based on data from Dai et al., 2017.

Supplementary Data 5. The .xlsx file includes the hierarchical clustering of different reactions based on their functional decomposition. The file includes two sheets: (1) Functional decomposition into energy production, AA and NT biosynthesis, and mixed function (the data underlying Fig. 4a). Only reactions with at least 50% of contribution to these functions ($\sum_\gamma F_i^{(\gamma)} > 0.5$) are shown. The table includes the assigned clusters obtained using two threshold values, 1 and 1.5 (see Methods). (2) Same as in the previous sheet, but for all metabolic functions and all active reactions.

Supplementary Data 6. The .xlsx file describes the demands of ATP and electron carriers associated to the biosynthesis of biomass precursors. See Supplementary Fig. 5j,k and Supplementary Note 4 for details.

Supplementary Data 7. The .xlsx file includes four sheets: (1) the growth rates and ATP yields obtained for growth under different carbon sources. (2) The flux modes associated to the oxidation of electron carriers (NADH, NADPH, FADH2, Q_8H_2) or to the leakage of protons from the periplasm to the cytoplasm, for the wild-type strain and the *nuo*$^-$ strain in glucose minimal media. The energy costs (encoded in the ATPM flux) determine the ATP equivalents for each electron carrier or for each proton. (3) Balance of ATP, cytoplasmic protons and electron carriers in the flux modes associated to

the biosynthesis of each biomass precursor. All reactions affecting their balance are shown with the corresponding production or consumption flux; each column sums up to zero. ATP maintenance reaction is highlighted in yellow, while the ATP synthetase (ATPS4rpp) and other reactions involved in electron transport are highlighted in orange. (4) Overall balance of ATP and electron carriers for all reactions in the previous sheet, excluding ATPM and electron transport chain reactions (highlighted in sheet 3). The net balance was converted into and effective ATP cost using the values in Supplementary Table 3. Comparison with the opposite of the ATPM flux in each flux mode shows a perfect agreement, implying that the conversion coefficient capture well the electron flow through the electron transport chain and their conversion into ATP via ATP synthetase.

Supplementary Data 8. The .xlsx file includes glucose and ATP costs, as well as protein allocation parameters (slopes and intercepts for the protein mass fractions, versus the demand flux for C- and R-limited growth) for individual amino acids and nucleotides.
