# Supplementary material for: Functional decomposition of metabolism allows a system-level quantification of fluxes and protein allocation towards specific metabolic functions
Source: Nat Commun. 2023 Jul 13;14:4161. doi: 10.1038/s41467-023-39724-7 (PMC10345195; doi:10.1038/s41467-023-39724-7)
Supplement: Supplementary file 12 — Reporting Summary [file 41467_2023_39724_MOESM12_ESM.pdf]

nature portfolio

Corresponding author(s): Matteo Mori

Last updated by author(s): 2023-05-31

Reporting Summary

Nature Portfolio wishes to improve the reproducibility of the work that we publish. This form provides structure for consistency and transparency in reporting. For further information on Nature Portfolio policies, see our Editorial Policies and the Editorial Policy Checklist.

Statistics

For all statistical analyses, confirm that the following items are present in the figure legend, table legend, main text, or Methods section.

n/a	Confirmed	
☐	☒	The exact sample size (*n*) for each experimental group/condition, given as a discrete number and unit of measurement
☐	☒	A statement on whether measurements were taken from distinct samples or whether the same sample was measured repeatedly
☒	☐	The statistical test(s) used AND whether they are one- or two-sided<br>*Only common tests should be described solely by name; describe more complex techniques in the Methods section.*
☒	☐	A description of all covariates tested
☒	☐	A description of any assumptions or corrections, such as tests of normality and adjustment for multiple comparisons
☐	☒	A full description of the statistical parameters including central tendency (e.g. means) or other basic estimates (e.g. regression coefficient) AND variation (e.g. standard deviation) or associated estimates of uncertainty (e.g. confidence intervals)
☒	☐	For null hypothesis testing, the test statistic (e.g. *F*, *t*, *r*) with confidence intervals, effect sizes, degrees of freedom and *P* value noted<br>*Give P values as exact values whenever suitable.*
☒	☐	For Bayesian analysis, information on the choice of priors and Markov chain Monte Carlo settings
☒	☐	For hierarchical and complex designs, identification of the appropriate level for tests and full reporting of outcomes
☒	☐	Estimates of effect sizes (e.g. Cohen's *d*, Pearson's *r*), indicating how they were calculated

Our web collection on statistics for biologists contains articles on many of the points above.

Software and code

Policy information about availability of computer code

Data collection	No software was used for data collection.
Data analysis	The FBA calculations were performed in Python (v3.9) using CVXPY (v1.3.1) and the GUROBI solver (GUROBIpy v9.5.2). COBRApy v0.26.3 was used to parse metabolic models, and additional analysis were performed in MATLAB R2015b.<br><br>The Python code necessary to run the functional decomposition on a given metabolic model is available on GitHub (https://github.com/ahoiching/FDM) and deposited on Zotero (Mori et al. 2023, see Code Availability statement).

For manuscripts utilizing custom algorithms or software that are central to the research but not yet described in published literature, software must be made available to editors and reviewers. We strongly encourage code deposition in a community repository (e.g. GitHub). See the Nature Portfolio guidelines for submitting code & software for further information.

Data

Policy information about availability of data

All manuscripts must include a data availability statement. This statement should provide the following information, where applicable:

- Accession codes, unique identifiers, or web links for publicly available datasets
- A description of any restrictions on data availability
- For clinical datasets or third party data, please ensure that the statement adheres to our policy

Modified iML1515 E. coli metabolic models with biomass composition tailored to specific growth conditions are provided in Supplementary Data 1. Growth rate and [illegible]

flux data obtained in this work are provided in Supplementary Data 2. The functional decomposition of reaction fluxes and proteins in various conditions are provided in Supplementary Data 3 and 4. Energy, carbon and protein costs of biosynthetic activities are provided in Supplementary Data 6 and 8.

Research involving human participants, their data, or biological material

Policy information about studies with human participants or human data. See also policy information about sex, gender (identity/presentation), and sexual orientation and race, ethnicity and racism.

Reporting on sex and gender	N/A
Reporting on race, ethnicity, or other socially relevant groupings	N/A
Population characteristics	N/A
Recruitment	N/A
Ethics oversight	N/A

Note that full information on the approval of the study protocol must also be provided in the manuscript.

Field-specific reporting

Please select the one below that is the best fit for your research. If you are not sure, read the appropriate sections before making your selection.

[X] Life sciences [ ] Behavioural & social sciences [ ] Ecological, evolutionary & environmental sciences

For a reference copy of the document with all sections, see nature.com/documents/nr-reporting-summary-flat.pdf

Life sciences study design

All studies must disclose on these points even when the disclosure is negative.

Sample size	For the flux measurements, no statistical methods were used to predetermine sample size. Sample sizes were chosen based on previous literature using similar experimental methods (Nature 528, 99–104 (2015)).
Data exclusions	No data was excluded from the study.
Replication	For the measurement of intake and outtake fluxes, the data were independently collected in growth limitation series, in which cells are grown in the same medium with a range of inducer or antibiotics concentrations. This experimental design leads to smooth variation in the data (growth rates and fluxes) as the growth is progressively limited, which allows to evaluate the data reproducibility from the observed scatter. The data were collected from independent cultures collected on the same day. Independent replicates were successfully performed on different days.
Randomization	As a non-clinical study, we did not perform randomization at the sample collection. Randomization is not relevant to this study since our data are grouped only under clear categories, such as pathways, nutrients, etc.
Blinding	We did not perform blinding at the sample collection. The quantitative measurements performed in this work are less susceptible to bias than qualitative experiments, thus eliminating the need of blinding procedures. Rather, all samples were treated similarly by the experimenters following a strict protocol for cell growth (see Methods) to ensure that cells are in steady state conditions at data collection, and thus maximizing reproducibility.

Reporting for specific materials, systems and methods

We require information from authors about some types of materials, experimental systems and methods used in many studies. Here, indicate whether each material, system or method listed is relevant to your study. If you are not sure if a list item applies to your research, read the appropriate section before selecting a response.

Materials & experimental systems

n/a	Involved in the study	
[X]	[ ]	Antibodies
[X]	[ ]	Eukaryotic cell lines
[X]	[ ]	Palaeontology and archaeology
[X]	[ ]	Animals and other organisms
[X]	[ ]	Clinical data
[X]	[ ]	Dual use research of concern
[X]	[ ]	Plants

Methods

n/a	Involved in the study	
[X]	[ ]	ChIP-seq
[X]	[ ]	Flow cytometry
[X]	[ ]	MRI-based neuroimaging
